# Quality of life in children receiving treatment for *Mycobacterium abscessus* otomastoiditis

**DOI:** 10.1111/coa.13931

**Published:** 2022-03-30

**Authors:** Theresa Y.S. Leow, Stijn Bekkers, Arno M. Janssen, Sjoert A.H. Pegge, Henricus P.M. Kunst, Jerome J. Waterval, Thijs T.G. Jansen, Stefanie S.V. Henriet, Koen J. van Aerde, Jakko van Ingen, Myrthe K.S. Hol

**Affiliations:** ^1^ Department of Otorhinolaryngology and Head and Neck Surgery Radboud University Medical Center Nijmegen The Netherlands; ^2^ Department of Medical Imaging Radboud University Medical Center Nijmegen The Netherlands; ^3^ Dutch Academic Alliance Skull Base Pathology Radboud University Medical Center Maastricht University Medical Center+ Nijmegen/Maastricht The Netherlands; ^4^ Department of Pediatric Infectious Disease and Immunology Amalia’s Children Hospital Radboud University Medical Center Nijmegen The Netherlands; ^5^ Radboudumc Center for Infectious Diseases Department of Medical Microbiology Radboud University Medical Center Nijmegen The Netherlands; ^6^ Department of Otorhinolaryngology and Head and Neck Surgery University Medical Center Groningen Groningen The Netherlands; ^7^ Research School of Behavioral and Cognitive Neurosciences Graduate School of Medical Sciences University of Groningen Groningen The Netherlands

**Keywords:** children, *Mycobacterium abscessus*, otomastoiditis, quality of life

**Key points**

- *Mycobacterium abscessus* is a multidrug‐resistant nontuberculous mycobacterium capable of causing otomastoiditis and its treatment is complex with frequent adverse events.
- Ten children (mean age: 8 years old; 70% males) were treated according to our institutional protocol.
- Patients within 1‐year post‐treatment reported a decreased QoL, with GCBI and COMBI scores of −12.91 and 10.94.
- In contrast, patients evaluated more than 1 year after finishing treatment reported an improved QoL by both the GCBI (difference (Δ) = 23.9 points; *p* > .05) and COMBI (Δ = 19.4 points; *p* = .00).
- The disease‐induced hearing loss is reduced with successful treatment.

## 1 INTRODUCTION

*Mycobacterium abscessus* (*M*. *abscessus*) is a rapidly growing nontuberculous mycobacterium (NTM), often dubbed the ‘antibiotic nightmare’ for its extensive resistance to antibiotics.^1^ Pulmonary infections, skin and soft tissue infections are its most frequent clinical manifestations, but otomastoiditis is also an established clinical entity.^2^ Treatment is difficult due to the multidrug resistance and lack of international consensus on therapeutic options as well as duration of treatment.

A previous study at our institute suggested a 6‐month antibiotic treatment strategy combining systemic and local treatment.^3^ This intensive, long‐term antibiotic regimen proved effective, but severely affects children's wellbeing. Children need a peripherally inserted central catheter (PICC) for long‐term intravenous antibiotics and both surgery and antibiotic treatment may induce hearing loss (HL). Adverse events (AE) such as nausea and vomiting, myelosuppression, toxic dermatitis and liver toxicity are frequent and the psychosocial impact of the treatment is thought to be the severe.^3^

Therefore, the assessment of quality of life (QoL) during and after treatment is important. This study aims to evaluate adverse events and QoL after treatment for *M*. *abscessus* otomastoiditis.

## 2 METHODS

### 2.1 Design

A retrospective analysis of QoL from patients treated for *M*. *abscessus* otomastoiditis at our referral centre was performed. Informed consent was obtained from the guardians and children above 12 years old. Guardians and patients were invited by phone. Upon informed consent, QoL questionnaires were sent by mail. The data were saved in an anonymised protected web‐based database. This study was approved by the independent regional ethics committee (CMO Arnhem‐Nijmegen; file number: 2020‐6257).

### 2.2 Participants

Patients treated between 2013 and January 2020 for *M*. *abscessus* otomastoiditis were eligible. The microbiological diagnosis was made based on auramine staining and culture on liquid (Mycobacterium growth indicator tubes; MGIT) and solid (Lowenstein–Jensen) media; drug susceptibility testing was performed by broth microdilution. Patients with insufficient data on presenting symptoms and treatment were excluded. We extracted baseline characteristics (age, gender and predisposing factors), date of diagnosis, onset and related symptoms, audiograms and radiological imaging, date and type of treatment interventions (i.e. surgery and antibiotics), and post‐treatment follow‐up from the electronic medical files. Radiological imaging was used as baseline for signs of progression or improvement of the disease. Also, imaging was screened for signs of complications (e.g. osteomyelitis, sinus thrombosis, meningitis or cerebritis).

### 2.3 Treatment

This treatment strategy consists of an intensive phase using intravenous imipenem‐cilastatin and tigecycline and oral treatment with azithromycin and clofazimine (Table 1). Topical treatment with imipenem‐cilastatin and tigecycline eardrops is added for the duration of tympanic membrane perforation. Surgical debridement is performed during the intensive phase. Surgery was performed to obtain cultures (in patients without diagnosis) and to reduce the local infection load by a mastoidectomy and atticotomy. In patients in whom the diagnosis was already confirmed, local antibiotics (imipenem/cilastatin 1 mg/ml, 1:1, and tigecycline 1 mg/ml) was also left in the mastoid cavity. In the more recent cases, ventilation tubes were removed (because of the risk of biofilm formation) and the tympanic membrane was perforated using a laser for subsequent topical treatment.

**TABLE 1:** Antibiotic protocol

Drug (dosage)	Duration	Side effects
Imipenem/cilastatin (60/60–100/100 mg/kg/day)	8 weeks	Nausea/vomiting Diarrhoea Neutropenia
Tigecycline (2.4 mg/kg/day)	8 weeks	Nausea/vomiting Anorexia Diarrhoea Liver test abnormalities Hypoalbuminemia
Clofazimine (50–100 mg/day)	24 weeks	Nausea/vomiting Diarrhoea QT‐prolongation Hyperlipidaemia
Azithryomycin (10 mg/kg/day)	24 weeks	Nausea/vomiting Anorexia Diarrhoea Rash QT‐prolongation
Topical imipenem/cilastatin (1 mg/ml, 1:1) and tigecyline (1 mg/ml)	In case of tympanic membrane perforation	–

Note

On day 1, all patients start with all antibiotics.

### 2.4 Quality of Life Measurement instruments

We applied the Glasgow Children's Benefit Inventory (GCBI) and the Chronic Otitis Media Benefit Inventory (COMBI) QoL questionnaires.^4^, ^5^ The GCBI is a validated retrospective questionnaire on QoL in children after treatment in paediatric otolaryngology.^6^, ^7^, ^8^ Patients and guardians are supposed to fill out the questionnaire together. It consists of 24 questions on the impact of certain treatment on various social and emotional aspects of a child's life.^5^, ^7^ A score of zero is considered as no change before/after treatment. Positive scores mirror good response while negative scores mean the condition has become worse.^5^ The four different domains (emotions, physical health, learning and vitality) are evaluated separately.^7^

The COMBI is a validated questionnaire to measure, in retrospect, the impact of otitis and related ear problems on QoL. It consists of 12 questions with 5‐scaled answers, in which a score higher than 38.5 means the condition has improved significantly. The Dutch version has also been validated and was used in this study.^4^

As both questionnaires do not control for cranial nerve involvement or whether hearing aids were required after intervention, we have added five questions on lasting AE’s to the questionnaires (Appendix S1).

### 2.5 Statistics

An independent *t*‐test was used to compare the QoL scores of patients who have finished the treatment regime <1 year ago and patients who have finished the same regimen at least one year ago. Pearson correlation or independent t‐test was used for possible confounders such as: gender, age, treatment duration, intravenous treatment duration and otorrhea recurrence during follow‐up.

## 3 RESULTS

**FIGURE 1:**
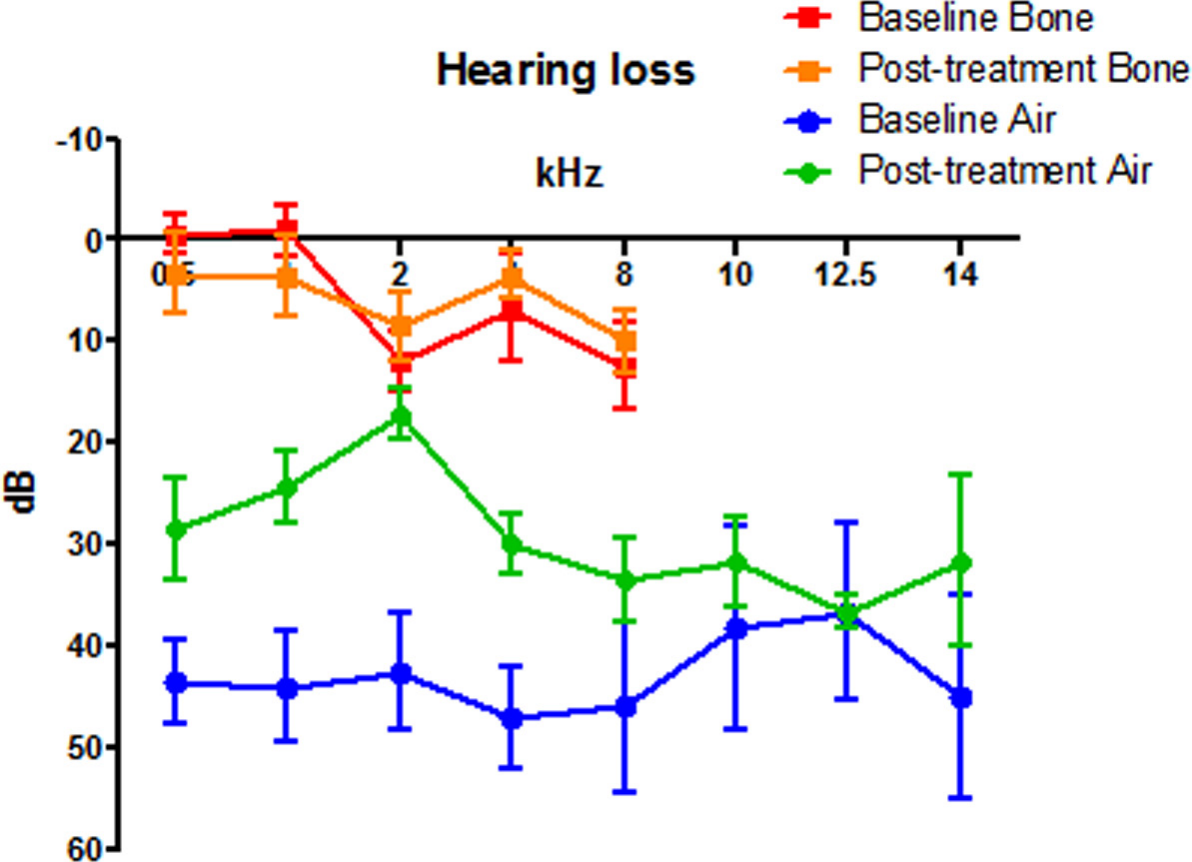
Mean hearing loss pre‐ and post‐treatment. Standard error of the means is shown using bars. Patient 6 has been included twice as both ears were affected

Ten patients were included. Patient characteristics are presented in Table 2. The actual duration per drug frequently deviated from protocol due to AEs and observed effect (see Figure S1). All patients suffered from conductive hearing loss (CHL) in the infected ear (Figure 1). On average a significant improvement of 26 dB was seen after treatment in the air‐bone gap in 0.5, 1.0, 2.0 and 4.0 kHz and bone conduction thresholds were unaffected. One patient suffered from azithromycin‐induced reversible sensorineural ototoxicity. In almost all patients, extensive granulation tissue was found in the mastoid cavity, attic, epitympanum, middle ear and external ear canal. All patients have finished treatment and improvement was seen during radiological, microbiological and clinical assessments. A mean follow‐up of 344 days (range 31–971 days) showed no recurrence of *M*. *abscessus* infection. Two patients had an ear infection (not NTM) and one patient reported an episode of otorrhea; all three clinically recovered.

**TABLE 2:** Patient characteristics

Case	Age (years), gender	Previous ear disease	Presdisposing factors	Side	Symptoms	Anatomical extension	Surgery and timing
001	7, M	ROM	VT, AB	Left	Ota, Oto, H, HL, TM	Middle ear and mastoid cavity	Tympanic tube removal (day 0)
							CAT; mastoidectomy and attico‐antrostomy including posterior tympanotomy. Removal of extensive granulation tissue from the mastoid and middle ear. (+4 weeks)
002	7, M	ROM, Adenotomy	VT, AB	Right	Oto, HL, S	Middle ear and mastoid cavity	CAT; mastoidectomy, attico‐antrostomy, epitympanotomy including posterior tympanotomy. Removal of extensive thickened middle ear mucosa. (+8 weeks)
003	15, F	ROM TM URTI	VT, AB	Left	Ota, Oto, HL	Middle ear and mastoid cavity	CAT; canalplasty, mastoidectomy and middle ear adhesiolysis (−20 weeks)
							Revision CAT; mastoidectomy. Removal of extensive thickened middle ear mucosa. (+3 weeks)
004	9, F	ROM URTI	History of VT, AB	Right	Oto, HL	Middle ear and mastoid cavity, carotid canal, petrous apex and infratemporal fossa. Enlarged retropharyngeal lymph nodes	CAT mastoidectomy. Removal of extensive thickened middle ear mucosa. (+5 weeks)
005	7, M	ROM Adenotomy Gradenigo syndrome	VT, AB	Right	Oto, H, F, V, N, P	Middle ear and mastoid cavity, petrous apex – clivus and part of the dura near the inner ear and fossa temporalis. Thickened Dorello canal, possibly suggesting inflammation of the abducens nerve as well	CAT; mastoidectomy (−2 weeks)
							CAT; mastoidectomy, attico‐antrostomy and epitympanotomy. Including posterior tympanotomy. (+8 weeks)
006	7, M	ROM TM	VT, AB	Both sides	Ota, Oto	Both sides: Middle ear, aditus antrum, mastoid cavity	CAT AS; mastoidectomy. Removal of extensive thickened middle ear mucosa. (−6 weeks)
							Revision CAT (both sides); mastoidectomy and attico‐antrostomy. Removal of extensive thickened middle ear mucosa. (+5 weeks)
							Revision mastoidectomy bilaterally (+14 weeks)
007	6, M	SOM	VT, AB	Right	Ota, Oto, H, F, T, WL	Middle ear and mastoid cavity, os petrosum, carotid canal and Eustachian tube. Parapharyngeal abscess. Dura and inner ear partly show enhancement. Thrombosis of the sigmoid sinus and transverse sinus until the jugular vein	Mastoidectomy and attico‐antrostomy. Removal of extensive middle ear granulation tissue (−1 week)
							Ear paracentesis (+8 weeks)
008	9, M	ROM, Oto	VT, AB	Left	Ota, Oto, HL, L	Middle ear and mastoid cavity	VT removal (−1 day)
009	5, F	ROM, Oto	VT, AB	Right	Oto, HL, TM	Middle ear and mastoid cavity. Enlarged lymph nodes in the neck area	Mastoidectomy and attico‐antrostomy. Removal of extensive middle ear granulation tissue. (+27 weeks)
010	8, M	ROM	VT, AB	Left	Ota, Oto, S, R	Middle ear and mastoid cavity, temporal bone and muscle. Slight dural enhancement on the lateral part of the temporal lobe. Enlarged lymph nodes	CAT; mastoidectomy and attico‐antrostomy. Removal of extensive middle ear granulation tissue (−1 day)

Note

Timing indicates the moment in which the surgical procedure was performed is relative to the start of the antibiotic treatment. Patient 008 did not undergo major surgery as radiological imaging showed the middle ear to be affected mostly with mild extension to the mastoid.

Abbreviations: AB, history of previous antibiotic treatment; CAT, Combined approach tympanoplasty; F, female; F, fever; H, headache; HL, hearing loss; L, lymphadenopathy; M, male; N, nausea; Ota, otalgia; Oto, otorrhea; P, photophobia; R, redness; ROM, recurrent otitis media; S, swelling; SOM, serous otitis media; T, tiredness; TM, tympanic membrane perforation; URTI, upper respiratory tract infection; V, vertigo; VT, ventilation tubes; WL, weight loss.

### 3.1 Quality of life

**FIGURE 2:**
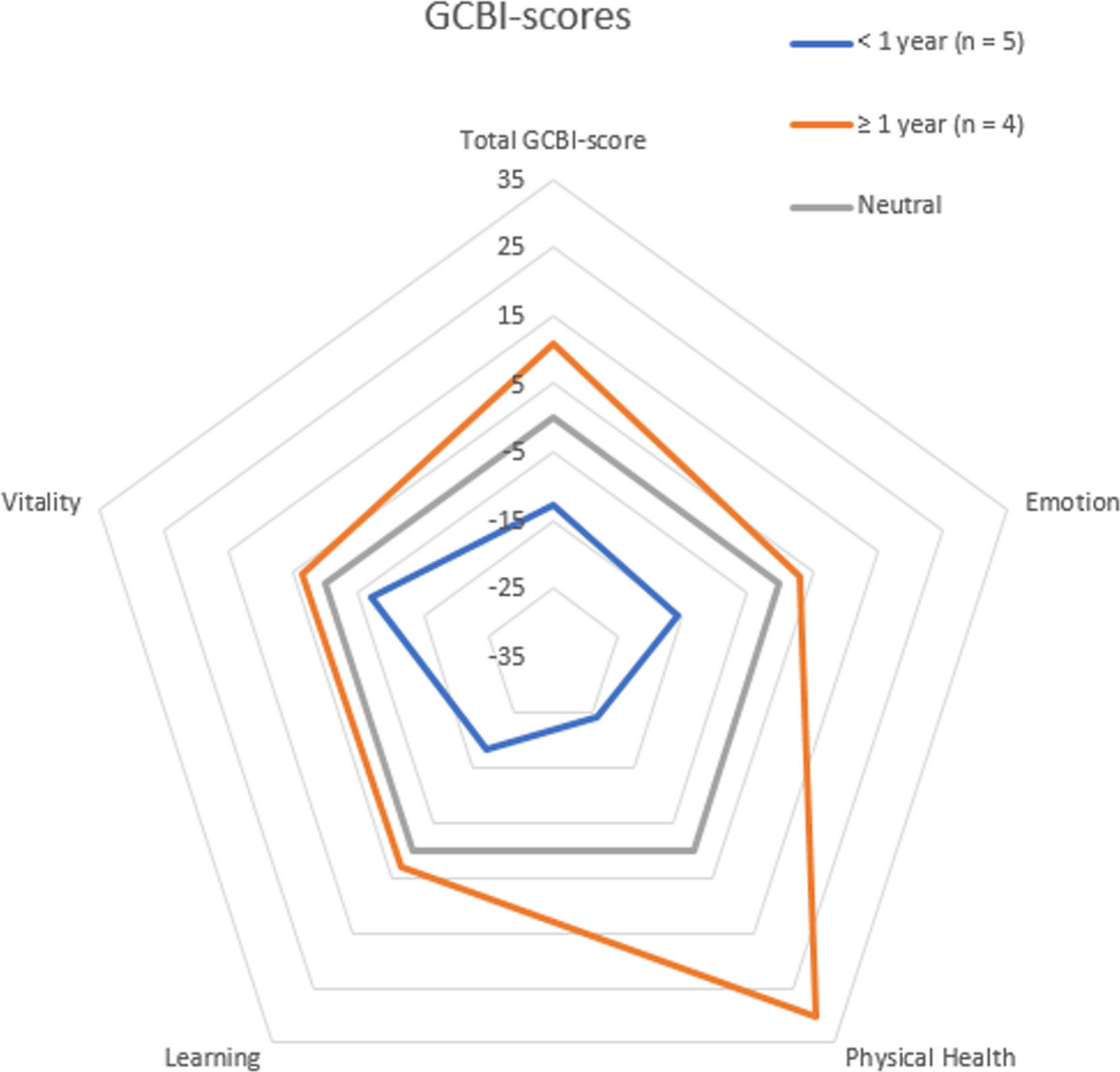
Spiderplot showing the GCBI‐score including subdomains. The population was divided in a group which finished treatment less than a year ago, and a group which finished treatment 1 year ago or longer. Baseline illustrates a GCBI‐score (=0) in which no change is perceive

Completed questionnaires were returned for nine children (90%). A total mean GCBI‐score of −2.3 (SD ± 19.8) was found. The total mean score and subdomains are presented in Figure 2. The mean COMBI score was 41 (±10.2). Age, gender, (iv) treatment duration and otorrhea recurrence did not impact significantly on COMBI or GCBI scores.

Patients who ended treatment less than 1 year ago had a lower mean GCBI‐score compared to patients who finished treatment more than 1 year ago ((−)12.9 vs. + 10.9; *p* = .991; 95% CI (−)52.3 to 4.6 [Figure 2]). This difference was significant in the subdomain physical health (∆ = 54.6; *p* = .005; 95% CI (−)86.1 – (−)23.2). Patients tested within 1 year after finishing treatment reported significantly lower COMBI scores than patients tested >1 year after starting/finishing antibiotic treatment (32.4 vs. 51.8; *p* < .001; 95% CI (−)25.6 – (−)13.1).

### 3.2 Post‐treatment AEs as reported by the children, parents or caregivers

Three patients (33.3%) suffer from subjective HL as reported by caregivers in the cross‐sectional survey, but bone conduction thresholds were unaltered. One patient (11.1%) reported tinnitus, weight gain and stretch marks (after dexamethasone usage). Nausea and dizziness after treatment were reported once (11.1%). Four caregivers (44.4%) emphasised the psychosocial effects, for example, concentration problem, emotional distress, anxiety and fear for hospitalisation. No cranial nerve palsies after treatment were reported.

## 4 DISCUSSION

This study evaluated ten patients who were treated for *M*. *abscessus* otomastoiditis and revealed a vast, but presumably temporary QoL reduction after treatment.

The GCBI showed relatively little improvement in QoL after treatment. The GCBI was previously used in children with Bone‐Anchored Hearing Aids who showed relatively great QoL improvement after implantation.^5^, ^9^ This may be due to side effects and long‐term nature of the treatment regimen. The GCBI‐scores also suggest that learning and emotion are the most affected by the treatment. Children will miss school for multiple weeks due to hospital admission(s) or AEs.

The COMBI showed a significant positive change post‐intervention on the physical and psychosocial impact of the chronic otitis media (COM), and our population scored similarly to COM patients who underwent surgery.^4^ The GCBI provides a broader view of the children's life in contrast to the COMBI which is specifically designed for COM. This could explain the difference in outcome: hearing and symptoms of infection improved significantly whereas in general, patients felt little positive change or even loss of quality of life.

Patients who finished treatment >1 year before completing GCBI and COMBI tests report significantly higher scores and an improved QoL compared to the most recently treated patients. This change over time after treatment cessation may have several possible reasons; (1) patients were still not recovered in the first year after treatment, (2) patients treated more recently had a more severe infection or disease spread, (3) time and the absence of disease recurrence may have mellowed negative feelings towards the intense treatment regime. Due to the small population size, a difference between severity of infection or AEs could not be evaluated. Retrospective questionnaires however, own a risk of bias related to the parents’ changed expectations over time and could partially explain the relative big improvement after 1 year post‐treatment.^7^

In lung infections by *M*. *abscessus*, where treatment approaches are similar albeit longer, a longitudinal study has shown QoL improvement with treatment, most evident in the first year. In the small cohort, QoL did not correlate with radiological or microbiological treatment outcomes.^10^ Another longitudinal study on NTM pulmonary infections showed worsening QoL during initiation of treatment and significant improvement after 1 year.^11^ Although the infection may be cured, patients may still suffer from side effects like affected hearing, fatigue, and psychosocial impact due to social isolation and school absence. We saw a gradual improvement of quality of life in time after curation. Based on this study and the experience of our patients, one year was chosen as criteria.

Besides recurrent ear infections, patients had no chronic illnesses or conditions which could have affected the QoL results. During the treatment period all patients were screened for primary immunodeficiencies and here, mainly humoral immune defects were found in a few cases. Also, these conditions did not affect the QoL per se but could of course have made patients more susceptible to infection.

The key limitations of the current study are the cohort size and the retrospective nature of the QoL assessments. As only 10 patients finished treatment and nine provided QoL data, statistical analysis is less reliable; conversely, a cohort this size with a standardised treatment for a rare but severe infection is unique and yet informative. One review only found 88 reported cases of NTM otomastoiditis in current literature, highlighting the rarity of this disease.^12^ A longitudinal study may have revealed the QoL in time better, nonetheless the instruments used are designed for retrospective analysis and provide important information.

## 5 CONCLUSION

In summary, antibiotic and surgical treatment of *M*. *abscessus* otomastoiditis led to a considerable, albeit presumably temporary decrease in the QoL. This should be conveyed to parents and patients when starting treatment for *M*. *abscessus* otomastoiditis. A longitudinal study should be performed to assess changes in QoL, as well as its predictors and its relation to treatment and outcome.

## CONFLICTS OF INTEREST

The authors have no relevant financial or non‐financial interests to disclose.

## AUTHORS’ CONTRIBUTIONS

TL, SB and MH designed the work; TL acquired and analysed data; TL drafted the manuscript, SB, MH, JvI, KvA, AJ, SP, HK, JW, TJ, SH revised and approved the manuscript. All agree to be accountable for all aspects of the work.

## ETHICAL APPROVAL

This study was performed in line with the principles of the Declaration of Helsinki. Approval was granted by the independent regional Ethics Committee of Arnhem‐Nijmegen (CMO) (Date:21‐02‐2020/No.2020‐6257).

## CODE AVAILABILITY

Not applicable.

## CONSENT TO PARTICIPATE

Informed consent was obtained from all individual participants included in the study.

## CONSENT FOR PUBLICATION

Not applicable.

[Correction added on May 31, 2022, after first online publication: Peer review history is not available for this article, so the peer review history statement has been removed.]

## Supporting information

Fig S1

Appendix S1

## Data Availability

Data may be available per request.
