# Supplementary figures and images for: Quality of life in children receiving treatment for Mycobacterium abscessus otomastoiditis
Source: Clin Otolaryngol. 2022 Mar 30;47(4):529–35. doi: 10.1111/coa.13931 (PMC9314591; doi:10.1111/coa.13931)

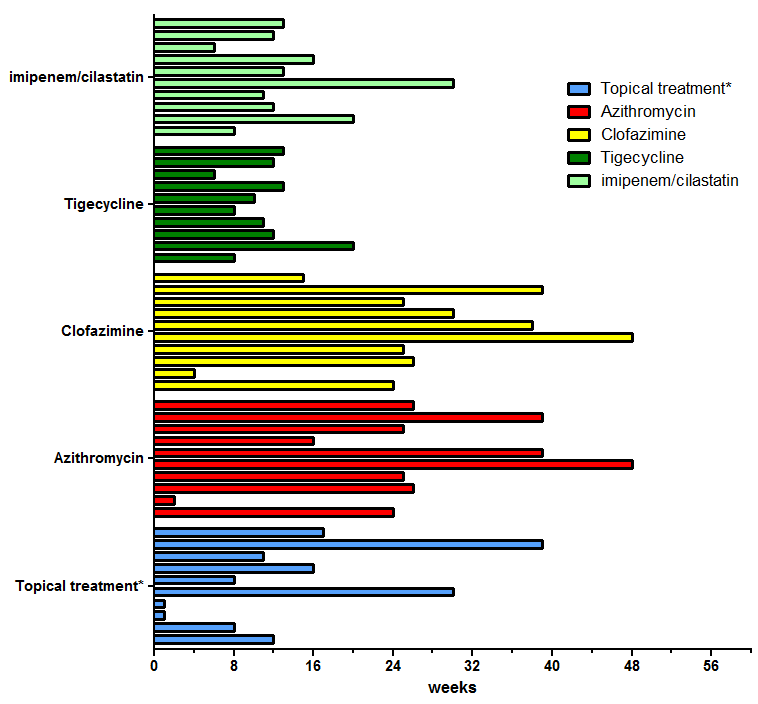

Supplement: Supplementary file 1 — Fig S1 [file COA-47-529-s002.tif]
