# Supplementary material for: Quality of life in children receiving treatment for Mycobacterium abscessus otomastoiditis
Source: Clin Otolaryngol. 2022 Mar 30;47(4):529–35. doi: 10.1111/coa.13931 (PMC9314591; doi:10.1111/coa.13931)
Supplement: Supplementary file 2 — Appendix S1 [file COA-47-529-s001.pdf]

Vragenlijsten voor kinderen na *Mycobacterium abscessus* otomastoiditis.

De vragenlijst bestaat uit drie delen. Het eerste deel (de Glasgow Children's Benefit Inventory) gaat met name over het algemene welzijn van uw kind. Het tweede gedeelte gaat met name over restverschijnselen na de behandeling van de infectie zijn overgebleven. Het derde deel gaat over de klachten van de oren. We maken gebruik van standaard vragenlijsten zodat we kunnen vergelijken met eerder onderzoek bij kinderen met oorklachten. Hierdoor kan zo zijn dat enkele vragen dubbel lijken voor te komen. Ondanks dat enkele vragen dubbel voor lijken voor te komen zouden wij u willen vragen om toch zo zorgvuldig mogelijk antwoord te geven op alle vragen. De vragenlijsten worden bij voorkeur door ouders met uw kind ingevuld.

Naast de vragen kunt u het cijfer dat correspondeert met uw antwoord omcirkelen.

Deel 1: Glasgow Children's Benefit Inventory

		Veel beter	Iets beter	Geen verschil	Iets slechter	Veel slechter
1.	Is het leven van uw kind sinds de behandeling in het algemeen beter of slechter geworden?	1	2	3	4	5
2.	Gaan de dingen die uw kind doet sinds de behandeling beter of slechter?	1	2	3	4	5
3.	Is het gedrag van uw kind sinds de behandeling beter of slechter?	1	2	3	4	5
4.	Zijn de vooruitgang en ontwikkeling van uw kind er sinds de behandeling beter of slechter op geworden?	1	2	3	4	5
5.	Is uw kind sinds de behandeling meer of minder levendig geworden?	1	2	3	4	5
6.	Slaapt uw kind beter of slechter sinds de behandeling?	1	2	3	4	5
7.	Is de eetlust van uw kind sinds de behandeling beter of slechter geworden?	1	2	3	4	5
8.	Voelt uw kind zich sinds de behandeling beter of slechter op zijn/haar gemakt bij andere mensen?	1	2	3	4	5
9.	Kan uw kind sinds de behandeling beter of slechter overweg met de rest van het gezin?	1	2	3	4	5
10.	Is uw kind sinds de behandeling beter of slechter in staat tijd door te brengen en plezier te maken met zijn/haar vriend(inn)en?	1	2	3	4	5
11.	Is uw kind sinds de behandeling meer of minder verlegen in het gezelschap van anderen?	1	2	3	4	5
12.	Is uw kind sinds de behandeling meer of minder makkelijk afgeleid?	1	2	3	4	5
13.	Leert uw kind beter of slechter sinds de behandeling?	1	2	3	4	5
14.	Is uw kind sinds de behandeling meer of minder afwezig geweest op het kinderdagverblijf, peuterspeelzaal of school?	1	2	3	4	5
15.	Kan uw kind zich sinds de behandeling beter of slechter op een taak concentreren?	1	2	3	4	5
16.	Is uw kind sinds de behandeling meer of minder gefrustreerd en geïrriteerd?	1	2	3	4	5
17.	Denkt uw kind sinds de behandeling beter of slechter over zichzelf?	1	2	3	4	5

		Veel beter	Iets beter	Geen verschil	Iets slechter	Veel slechter
18.	Is uw kind sinds de behandeling meer of minder gelukkig en tevreden?	1	2	3	4	5
19.	Is het zelfvertrouwen van uw kind sinds de behandeling beter of slechter geworden?	1	2	3	4	5
20.	Kan uw kind sinds de behandeling beter of slechter voor zichzelf zorgen, zoals zichzelf wassen, aankleden en naar de wc gaan?	1	2	3	4	5
21.	Kan uw kind sinds de behandeling beter of slechter genieten van vrijetijdsbestedingen zoals sporten en spelen?	1	2	3	4	5
22.	Is uw kind sinds de behandeling meer of minder vatbaar voor verkoudheden en ontstekingen?	1	2	3	4	5
23.	Moet uw kind sinds de behandeling meer of minder vaak naar een arts?	1	2	3	4	5
24.	Heeft uw kinds sinds de behandeling meer of minder medicijnen nodig?	1	2	3	4	5

Deel 2: vragenlijst over eventuele restverschijnselen van de behandeling

		Veel minder	Iets minder	Geen verschil	Iets meer	Veel meer	N.v.t.
1.	Sinds de behandeling, heeft uw kind meer of minder last van hoofd of aangezichtspijn?	1	2	3	4	5	0
2.	Sinds de behandeling, is uw kind meer of minder scheel gaan kijken?	1	2	3	4	5	0
3.	Sinds de behandeling, heeft uw kind een aangezichtsverlamming gekregen?	Ja	Nee				
**4.**	Indien u ja hebt geantwoord bij vraag 4, is deze aangezichtsverlamming meer of minder geworden?	1	2	3	4	5	0
5.	Heeft uw kind na de behandeling een gehoortoestel gekregen?	Ja	Nee				
6.	Zijn er andere restverschijnselen na de behandeling overgebleven? Graag hier specificeren: …… …… …… …… …………………………………………………						

Tot slot zouden wij u willen vragen of u nog andere opmerkingen heeft ten aanzien van het ziekte proces of de behandeling van deze infectie:

……
……
……
……

***Referentie#

Deel 3: Chronic Otitis Media Benefit Inventory

Onderstaande vragen zijn om te achterhalen hoe erg de oorproblemen uw kind beïnvloeden in vergelijking met de situatie voor de operatie/behandeling. Geen enkele machine kan dit voor u doen: enkel u en uw kind kunnen ons dit vertellen. Deze wetenschap zal ons helpen om de wijze waarop patiënten met oorproblemen door mycobacterium abscessus worden verzorgd te verbeteren.

Beantwoord alstublieft onderstaande vragen zorgvuldig door elke gestelde vraag te overwegen en vervolgens het geschikte cijfer te omcirkelen. De cijfers verwijzen elk naar een bepaalde beschrijving die eronder vermeldt staat. Er zijn geen juiste of foute antwoorden, maar probeert u alstublieft goed na te denken over elke vraag voordat u het geschikte cijfer omcirkelt.

Ernst van de symptomen

1. Sedert uw operatie/behandeling, is uw oorloop of drainage van uw oor verbeterd of verslechterd?

5	4	3	2	1
Veel beter	Een beetje beter	Onveranderd	Een beetje slechter	Veel slechter

2. Sedert uw operatie/behandeling , hoe zou u de verandering beschrijven in het hebben van een 'slecht ruikend oor'? Is dit verbeterd of verslechterd?

5	4	3	2	1
Veel beter	Een beetje beter	Onveranderd	Een beetje slechter	Veel slechter

3. Sedert uw operatie/behandeling, is uw gehoor thuis (bijv. de televisie of de radio luider moeten zetten) verbeterd of verslechterd?

5	4	3	2	1
Veel beter	Een beetje beter	Onveranderd	Een beetje slechter	Veel slechter

4. Sedert uw operatie/behandeling, is uw gehoor wanneer u met anderen in groep spreekt (of wanneer u in een lawaaierige omgeving bent) verbeterd of verslechterd?

5	4	3	2	1
Veel beter	Een beetje beter	Onveranderd	Een beetje slechter	Veel slechter

5. Sedert uw operatie/behandeling, is het discomfort in en/of rond het oor verbeterd of verslechterd?

5	4	3	2	1
Veel beter	Een beetje beter	Onveranderd	Een beetje slechter	Veel slechter

6. Sedert uw operatie/behandeling, is uw duizeligheid of uw gevoel van 'instabiliteit' verbeterd of verslechterd?

5	4	3	2	1
Veel beter	Een beetje beter	Onveranderd	Een beetje slechter	Veel slechter

7. Sedert uw operatie/behandeling, is uw tinnitus of lawaai in het oor verbeterd of verslechterd?

5	4	3	2	1
Veel beter	Een beetje beter	Onveranderd	Een beetje slechter	Veel slechter

z.o.z.

Gevolgen voor levensstijl, werk/school en gezondheidszorg

8. Betreffende uw gewone dagelijkse activiteiten thuis en op school, zou u zeggen dat u meer problemen of minder problemen ondervindt, sedert uw operatie/behandeling?

1	2	3	4	5
Veel meer problemen om activiteiten uit te voeren	Meer problemen om activiteiten uit te voeren	Onveranderd	Meer problemen om activiteiten uit te voeren	Veel minder problemen om activiteiten uit te voeren

9. Betreffende de mogelijkheid om u te wassen of te douchen of te baden zoals u zelf zou willen sedert uw operatie/behandeling, hebt u dan meer angst of minder angst om een oorontsteking te krijgen door deze activiteiten?

1	2	3	4	5
Veel meer angst dat het oor nat wordt	Meer angst dat het oor nat wordt	Onveranderd	Minder angst dat het oor nat wordt	Veel minder angst dat het oor nat wordt

10. Sedert uw operatie/behandeling, bent u vaker of minder vaak naar uw huisarts gegaan omwille van uw oorproblemen?

1	2	3	4	5
Veel vaker	Vaker	Onveranderd	Minder vaak	Veel minder vaak

11. Sedert uw operatie/behandeling, heeft u vaker of minder vaak medicijnen (met inbegrip van oordruppels) moeten nemen voor uw oorprobleem?

1	2	3	4	5
Veel vaker	Vaker	Onveranderd	Minder vaak	Veel minder vaak

Algemeen

12. Sedert uw operatie/behandeling, bent u meer of minder ‘onderuit gehaald’ door uw oorprobleem dan ervoor?

1	2	3	4	5
Veel meer dan voorheen	Meer dan voorheen	Onveranderd	Minder dan voorheen	Veel minder dan voorheen

Hartelijk dank voor het invullen van de vragenlijsten!

***Referentie#
